# Association Between Postoperative Symptom Severity and Oral Health-Related Quality of Life After Mandibular Third Molar Extraction

**Published:** 2026-04-10

**Authors:** NOORULAIN FATIMA, EKRA RAJ, KHUDEMA MOEEN, ABDULLAH MAQSOOD, WALEED AHMED, SARA INAYAT, ZEESHAN ALVI, RIDA MEHBOOB ELLAHIE, FATIMA RIZWAN, MARYAM SAJID, MUHAMMAD SAAD BIN WARIS

**Affiliations:** 1 Department of Dentistry, Shifa College of Dentistry, Rawalpindi, Pakistan; 1 Department of Dentistry, Shifa College of Dentistry, Rawalpindi, Pakistan; 2 Department of Dentistry, Foundation University College of Dentistry, Islamabad, Pakistan; 2 Department of Dentistry, Foundation University College of Dentistry, Islamabad, Pakistan; 3 Department of Dentistry, Punjab Dental Hospital, Lahore, Pakistan; 3 Department of Dentistry, Punjab Dental Hospital, Lahore, Pakistan; 4 Department of Public Health, York St John University, London, United Kingdom; 4 Department of Public Health, York St John University, London, United Kingdom; 5 Department of Dental Surgery, European University of Lefke, Lefke, Cyprus; 5 Department of Dental Surgery, European University of Lefke, Lefke, Cyprus; 6 Department of Dentistry, Watim Medical and Dental College, Rawalpindi, Pakistan; 6 Department of Dentistry, Watim Medical and Dental College, Rawalpindi, Pakistan

**Keywords:** mandibular third molar extraction, postoperative symptom severity, oral health-related quality of life, PoSSe scale, OHIP-14

## Abstract

**Objectives:**

Third molar extraction of the mandible is considered to be among the most common forms of oral surgery, which is accompanied by post-surgery pain, swelling, and functional disabilities. The symptoms may affect patients' Oral health-related quality of life (OHRQoL). This research investigated the correlation between postoperative symptom severity and OHRQoL in mandibular third molar removal.

**Materials and Methods:**

A cross-sectional study was conducted among 386 patients who presented to the oral and maxillofacial surgery outpatient departments. A structured questionnaire containing demographic data, the Postoperative Symptom Severity (PoSSe) scale, and the Oral Health Impact Profile (OHIP-14) was used to gather data. Pearson correlation, t-tests, one-way ANOVA, and multiple linear regression were used to identify predictors of OHRQoL.

**Results:**

The average scores of PoSSe and OHIP-14 were 41.0 and 42.2, respectively. A strong positive correlation was found between PoSSe and OHIP-14 scores (r = 0.58, *p* < 0.001), indicating lower OHRQoL as symptom severity increased. Women scored significantly higher than men on both PoSSe and OHIP-14 scales (*p* < 0.001). People aged 56 years and above indicated the highest symptom severity and poorest OHRQoL. PoSSe was the most potent indicator of OHRQoL (B = 0.420, *p* < 0.001), with age and sex following next.

**Conclusions:**

The severity of the postoperative symptoms is a significant factor in defining OHRQoL after mandibular third molar extraction. The findings demonstrate the significance of adopting an individualised postoperative pain management, providing patient education, and delivering personalised postoperative support to improve recovery rates.

## Introduction

Mandibular third molar extraction is commonly performed in oral and maxillofacial surgery, often due to pericoronitis, caries, tissue destruction, or other associated cysts and tumours^1)^. Although most complications are not serious, the patient's age and the need to excise bone can contribute to adverse outcomes. That is why the organisation of surgery should be cautious^2)^.

One of the potential complications that may arise is injury to the inferior alveolar nerve, and its incidence is reported to be 0.35-8.4%^3)^. Perioperative oral prednisolone 20mg or less has not been demonstrated to have a significant reduction of postoperative symptoms^4)^.

Patient-related factors, rather than surgical factors, were the main contributors to postoperative discomfort following mandibular third molar extraction^5)^. The Postoperative Symptom Severity (PoSSe) scale has also been developed to evaluate the experiences of patients who have undergone third molar extraction and has been observed to be reliable, valid, and responsive^6)^.

The extraction of lower third molars may also substantially affect patients' quality of life (QoL), especially during the initial postoperative days. However, its impact tends to wane and return to baseline after one week^7, 8)^. Acute pain, trismus, and swelling are usually at their highest levels on the first day after the operation and fade over the week, as reported by the OHIP-14 questionnaire^9)^.

The relationship between the intensity of postoperative symptoms and OHRQoL needs to be comprehended since transient pain can affect the usual daily way of life, socialisation, and well-being. Although the nature of individual postoperative symptoms has been studied extensively, as described earlier, few have been measured in terms of their combined impact on OHRQoL following mandibular third molar surgery. The objectives of the research are thus to determine the correlation between the severity of postoperative symptoms and quality of life following removal of the mandibular third molar, in relation to oral health. This relationship can help clinicians better predict patient demand, maximise post-surgery care, and deliver patient-centred outcomes.

### Aims

The purpose of the study is to determine the relationship between the severity of postoperative symptoms and OHRQoL among patients undergoing removal of mandibular third molars. The objective of the study is to examine the effect of the cumulative burden of postoperative symptoms on perceived oral health among patients, while controlling for demographics such as age and gender. This relationship would enable the research to furnish information to guide the practice of patient-centred postoperative care and to improve postsurgical recovery after third molar surgery.

### Research design and methods

It was a cross-sectional study that established the relationship between the intensity of postoperative symptoms and OHRQoL in patients who underwent removal of the mandibular third molar. The study sampled outpatient oral and maxillofacial surgery departments in both public and private clinics and obtained written or oral informed consent from participants in accordance with ethical guidelines.

### Sampling strategy and population size

The sample size was determined on the assumption that the population is infinite, the prevalence (p) = 0.5, the local data is unavailable, the confidence level (Z = 1.96), and a 0.05 margin of error. Patients were recruited from the oral and maxillofacial surgery outpatient units of selected hospitals in Islamabad and Rawalpindi (public and private) using convenience sampling. A total of 420 patients were approached, of whom 34 responded that they were ineligible or declined to participate^10)^. The other 386 patients gave informed consent and were included in the final analysis.

Although convenience sampling is adequate for recruiting and allocating resources, it can introduce selection bias, limiting the generalizability of results to patients outside the chosen clinics.

### Inclusion and exclusion criteria

The eligibility criteria for this study were to identify adults aged 18 and above who had undergone mandibular third molar extraction. They had follow-up visits at the participating oral and maxillofacial surgery clinics. Only persons willing and capable of providing written or oral informed consent were included. Any systemic condition that could affect healing or the perception of pain (uncontrolled diabetes or immunodeficiency) or medications that could alter postoperative symptoms (long-term corticosteroids or chronic analgesics) were excluded. Additionally, patients with a history of mandibular surgery before the study, problems in the third molar region, or who could not undergo the study procedures due to cognitive impairment or language barriers were excluded from the study.

### Data collection tools

A structured questionnaire was designed to collect relevant information for this study, including three sections: demographics, postoperative symptom severity, and OHRQoL. All the tools were applied in their original language, that is, English, without any language or cultural adaptations.

The demographic and clinical data were collected in the first part of the questionnaire to enable subgroup analysis and to investigate possible relationships with postoperative outcomes. Variables included age, gender, and pertinent medical history, including comorbidities and prior oral surgical visits.

### Postoperative symptom severity (PoSSe) questionnaire

In 2000, Danny Ruta (E. Bissias, S. Ogston, and G.R. Ogden) created the PoSSe Questionnaire to measure the intensity of postoperative symptoms after third molar extraction. The initial version of the scale comprised 15 items assessing components of postoperative recovery, including pain, swelling, trismus, and difficulty eating. Item scores are determined on a Likert scale, and higher total scores indicate more severe overall symptoms^6)^. This research study employed the PoSSe to measure patients' postoperative symptom burden and the relationship between this burden and oral health as a QoL indicator.

### Oral health impact profile (OHIP-14)

In 1997, Slade created the OHIP-14, a shorter version of the original 49-item measure, to assess OHRQoL. It has 14 questions across seven domains, rated on a 5-point Likert scale (0 = never, 4 = very often), with higher scores indicating a greater adverse effect on quality of life. In the study, the OHIP-14 was administered to assess the impact of postoperative symptoms on daily functioning and overall OHRQoL^11)^.

### Procedure

Participants were recruited through written or oral informed consent of patients visiting the oral and maxillofacial surgery outpatient departments. The data were collected over 5 months (April 2025 to October 2025). The patients who had undergone mandibular third molar extraction were approached by the trained research assistants after their follow-up visits, and the purpose of the study was explained to them.

Follow-up visits were conducted regularly, during which trained research assistants approached patients who had undergone mandibular third molar extraction and informed them of the study. Both the PoSSe scale and the OHIP-14 were administered at a standardized postoperative time point, seven days after mandibular third molar extraction, to ensure uniform assessment of symptom severity and oral health-related quality of life. The structured questionnaire was completed either by self- administration or face-to-face, according to participants' literacy level and preference. For participants who were not fluent in English, standardized verbal translation assistance was provided by trained research assistants to ensure consistent interpretation of questionnaire items across all respondents. To ensure that the study data did not compromise participants' confidentiality, the data were anonymised, and their identities were protected. The research was conducted in a responsible, just, and inclusive manner, allowing participants to reflect on diverse demographic and socioeconomic groups. The methodology helped create a representative sample of postoperative experiences and OHRQoL outcomes, while also accounting for the limitations of convenience sampling.

### Statistical analysis and ethical considerations:

Statistical analysis was performed using SPSS version 26. The demographic variables were the PoSSe and OHIP scores, and the descriptive statistics summarised them using means, standard deviations, frequencies, and percentages. Pearson correlation was used to determine the relationship between PoSSe and OHIP scores. Gender comparisons were conducted using independent-samples t-tests, whereas age group differences were compared using one-way ANOVA. Significant predictors of postoperative OHIP scores, including postoperative symptoms, demographic factors, and clinical characteristics, were identified using multiple linear regression analysis. The p-value was less than 0.05, indicating significance.

The study was approved by the Institutional Review Board of Shifa College of Dentistry, Rawalpindi, Pakistan (SCD-IRB/REC-DENT/2025-04- 021), and informed consent was obtained from all participants through written/oral means. Participants could withdraw, and the data were anonymised to ensure confidentiality. The study was conducted in compliance with the Declaration of Helsinki, and ethical, respectful, and culturally sensitive relationships were maintained with all respondents.

## Results

### Participant demographic and clinical characteristics

Table 1 summarises the participants' properties (N = 386). The majority were between 18 and 25 years old (N = 150, 38.9%), with females comprising the majority (N = 221, 57.3%). The largest work groups were employed (N = 112, 29.0%) and self- employed (N = 110, 28.5%). The most common were primary (N = 145, 37.6%) and secondary education (N = 99, 25.6%). Almost half of them were non- smokers (N = 177, 45.9%). The most common extraction was of the right mandibular third molar (N = 202, 52.3%), and an extraction of partial bony impaction was the most prevalent (N = 147, 38.1%). The mean duration of most procedures was 15-30 minutes (N = 154, 39.9%), and most had prior extraction experience (N = 251, 65.0%). Over half of the participants took postoperative medications irregularly (N = 218, 56.5%).

**Table 1:** Demographic characteristics of participants (N = 386)

Variable	f	%		Variable	f	%
Age				Smoking status		
18-25 years	150	38.9		Non-smoker	177	45.9
26-35 years	95	24.6		Former smoker	122	31.6
36-45 years	60	15.5		Current smoker	87	22.5
46-55 years	45	11.7		Side of extraction (mandibular third molar)		
56 years or above	36	9.3		Left	58	15.0
Gender				Right	202	52.3
Male	165	42.7		Both sides	126	32.6
Female	221	57.3		Type of impaction (as told by dentist)		
Marital status				Soft tissue impaction	73	18.9
Student	75	19.4		Partial bony impaction	147	38.1
Employed	112	29.0		Complete bony impaction	120	31.1
Self-employed	110	28.5		Don't know	46	11.9
Homemaker	58	15.0		Surgical procedure duration		
Unemployed	21	5.4		< 15 minutes	50	13.0
Other	10	2.6		15-30 minutes	154	39.9
Educational level				31-45 minutes	132	34.2
No formal education	71	18.4		> 45 minutes	50	13.0
Primary	145	37.6		Previous third molar extraction experience		
Secondary	99	25.6		Yes	251	65.0
Higher secondary	35	9.1		No	135	35.0
Graduate	23	6.0		Use of postoperative medications		
Postgraduate	13	3.4		Yes, regularly	92	23.8
				Yes, irregularly	218	56.5
				No	76	19.7

Note. N = number of participants; f = frequency, % = percentage; N = 386

### Correlation between postoperative symptom severity and oral health-related quality of life

Table 2 shows a strong positive correlation between Postoperative Symptom Severity (PoSSe) and Oral Health Impact Profile (OHIP-14) scores (r = 0.58, N = 386, *p* < 0.001), indicating that greater postoperative symptom severity is associated with poorer oral health-related quality of life.

**Table 2:** Pearson correlations among postoperative symptom severity (PoSSe), and oral health impact profile (OHIP) scales (N = 386)

Variable	1	2
Postoperative symptom severity (PoSSe)	-	r = 0.58, t = 13.95, p =< 0.001***
Oral health impact profile (OHIP)	-	-

Note. ***=p < 0.001 considered significant; Positive correlations indicate direct relationships; N = 386.

### Gender-based differences in postoperative symptom severity and OHRQoL

Table 3 presents a comparison of gender differences in PoSSe and OHIP among the study subjects (N = 386), with 165 males (42.7%) and 221 females (57.3%). Females scored higher on PoSSe than males (t(384) = 6.23, *p* < 0.001***, 95% CI = 2.39-4.61, Cohen’s d = 0.86). Similarly, females had higher OHIP scores than males (t(384) = 5.14, *p* < 0.001***, 95% CI = 1.85-4.15, Cohen’s d = 0.66). These results indicate that females reported more postoperative symptoms and a greater impact on oral health than their male counterparts.

**Table 3:** Gender differences in PoSSe and OHIP scores (N = 386)

Variables	Male (N=165; 42.7%);(M ± SD)	Female (N=221; 57.3%); (M ± SD)	*t*	*p*	95% CI LL	95% CI UL	Cohen's *d*
Postoperative symptom severity (PoSSe)	39.0 ± 4.0	42.5 ± 4.2	6.23	<0.001***	2.39	4.61	0.86
Oral health impact profile (OHIP)	40.5 ± 4.5	43.5 ± 5.0	5.14	<0.001**	1.85	4.15	0.66

Note. Values are presented as Mean ± Standard Deviation; Independent samples t-tests were conducted to compare participants of both male and female groups; Group sizes are shown as N (%); Reported statistics include p-values, t-values, 95% Confidence Intervals (CI), and effect sizes (Cohen's d); A p-value < 0.001 was considered statistically significant, N = 386.

### Age-related differences in postoperative symptom severity and OHRQoL

Table 4 presents age-specific modifications of the PoSSe and OHIP scores in the sample (N = 386). The scores of PoSSe rose steadily with age, with 39.0 ± 4.0 among the participants in the 18-25 years of age (N = 150; 38.9) and 44.0 ± 4.7 in the 56 and above years (N = 36; 9.3), and there was significant overall effect, F(4, 381) = 7.14, p = 0.001, 2 = 0.069. Similarly, the OHIP scores increased with age, with a mean score of 40.5 ± 4.5 in the youngest group and 46.0 ± 5.3 in the oldest group, and the significant group difference was 8.92 (p = 0.001, 0.085). These results indicate that the older participants experienced more severe postoperative symptoms and oral health effects compared to the younger participants.

**Table 4:** Descriptive statistics and One-way ANOVA of postoperative symptom severity (PoSSe) and oral health impact profile (OHIP) across age groups (N = 386)

Variables	18-25 years (N = 150; 38.9%); (M ± SD)	26-35 years (N = 95; 24.6%); (M ± SD)	36-45 years (N = 60; 15.5%); (M ± SD)	46-55 years (N = 45; 11.7%); (M ± SD)	56+ years (N = 36; 9.3%); (M ± SD)	F(4,381)	p	η^2^
Postoperative symptom severity (PoSSe)	39.0 ± 4.0	41.5 ± 4.2	42.0 ± 4.3	43.0 ± 4.5	44.0 ± 4.7	7.14	< 0.001***	0.069
Oral health impact profile (OHIP)	40.5 ± 4.5	43.0 ± 5.0	44.0 ± 5.1	45.5 ± 5.2	46.0 ± 5.3	8.92	< 0.001***	0.085

Note. Data are presented as Mean ± Standard Deviation (M ± SD); Group sizes are shown as N (%). One-way ANOVA was conducted to examine the effect; All comparisons were significant at *p* <0.001; η^2^ represents the partial eta-squared effect size.

### Predictors of oral health-related quality of life: multiple linear regression analysis

Table 5 presents the multiple linear regression predicting Oral Health Impact Profile (OHIP) scores for participants (N = 386). Positive predictors of OHIP scores showed the strongest and most significant positive correlation with Postoperative Symptom Severity (PoSSe) (B = 0.420, 0.435; t = 7.636; p = 0.001), indicating that the more severe the symptoms, the greater the negative impact of poor oral health. There was also a significant positive relationship between age (B = 0.315, p = 0.004), indicating that older age was associated with higher OHIP scores. Females had much higher OHIP scores than males (B = 1.950, *p* < 0.001). Also, the type of impaction (B = 0.734, p = 0.002) and side of extraction (B = 0.512, p = 0.016) were significant predictors, with more complex extraction characteristics associated with poorer OHIP outcomes. On the other hand, previous extraction experience (B = -1.120, p = 0.001) and postoperative medication use (B = -0.682, p = 0.010) were negative predictors, and OHIP scores were lower among individuals with previous extraction experience and among those who had used postoperative medication. In general, the model identifies several demographic and clinical determinants that influence postoperative oral health quality of life.

**Table 5:** Multiple linear regression predicting oral health impact profile (OHIP) scores (N = 386)

Predictor	B	SE B	Beta	t	p	95% CI Lower	95% CI Upper
(Constant)	35.210	2.421	-	14.545	< 0.001***	30.426	40.0
Postoperative symptom severity (PoSSe)	0.420	0.055	0.435	7.636	< 0.001***	0.312	0.528
Age	0.315	0.108	0.142	2.917	0.004**	0.102	0.528
Gender (Male = 1, Female = 2)	1.950	0.532	0.175	3.667	< 0.001***	0.902	2.998
Side of extraction (mandibular third molar)	0.512	0.210	0.092	2.438	0.016*	0.096	0.928
Type of impaction	0.734	0.232	0.125	3.163	0.002**	0.278	1.190
Previous extraction experience (Yes = 1, No = 2)	-1.120	0.345	-0.145	-3.246	0.001**	-1.798	-0.442
Use of postoperative medications (Yes = 1, No = 2)	-0.682	0.261	-0.102	-2.611	0.010**	-1.195	-0.169

Note. Multiple linear regression was conducted to identify predictors of Oral Health Impact Profile (OHIP) Scores; Values include unstandardized coefficients (B), 95% confidence intervals (CI), standard error (SE), standardized beta coefficients (β), and p-values; *= *p* < 0.05, **= *p* < 0.01, ***= *p* < 0.001 was considered statistically significant, N = 386.

### Association between side of mandibular third molar extraction and postoperative medication Use

Figure 1 demonstrates a significant correlation between the side of tooth removal and the use of postoperative medications (χ^2^ = 48.73, *p* < 0.001). Among patients with left-side extractions, almost half (48.30%) did not take medication, and 17.20% did so regularly. Conversely, most of the people whose extractions were on the right (74.3) were taking such medications irregularly, with only very few (1) not taking any medication. Medication use was more equal on both sides in extractions: 25.4% and 38.5% reported regular and no use, respectively. This implies that the extraction side plays a significant role in determining the patterns of medication use.

**Figure 1:**
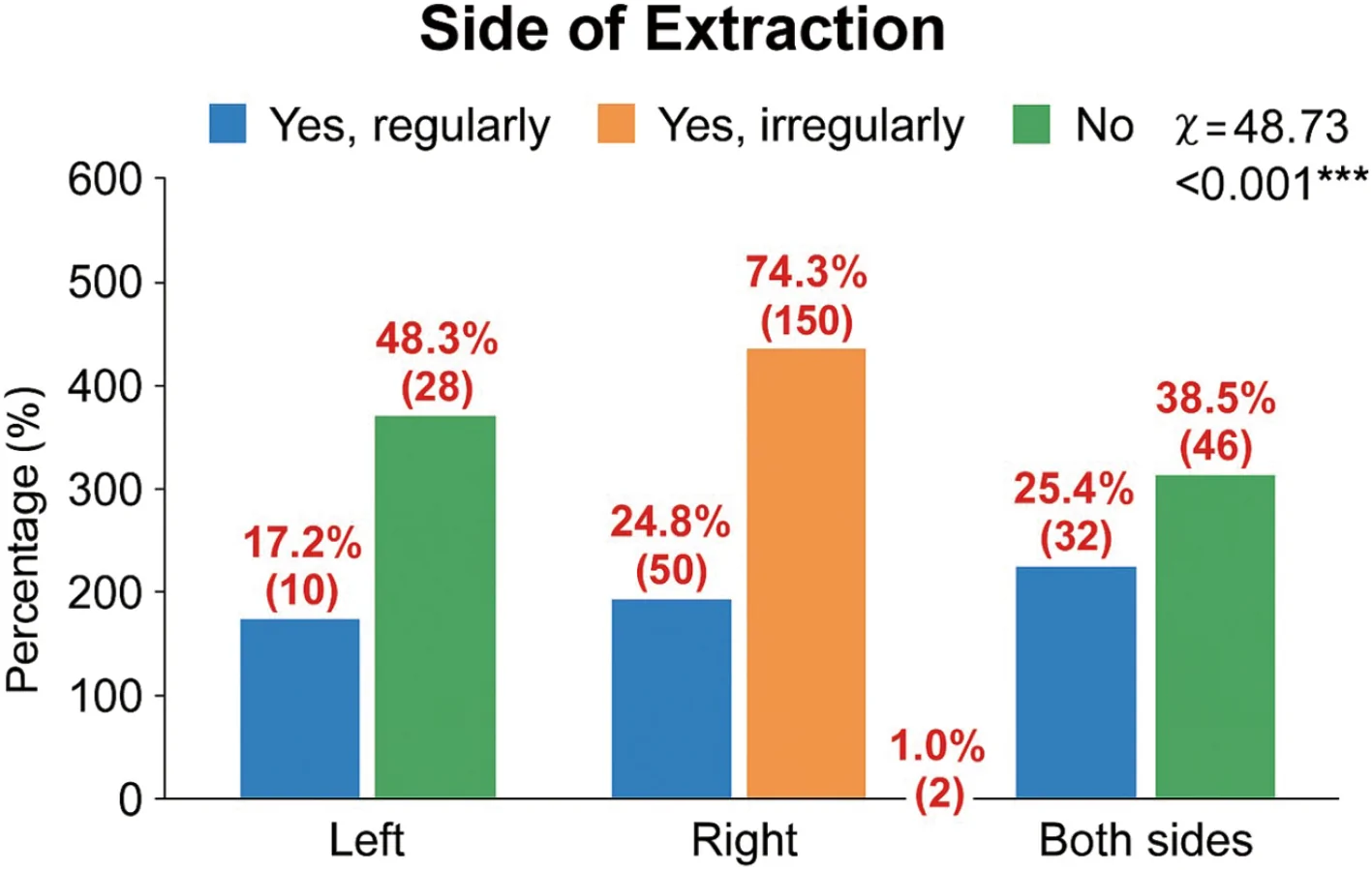
Distribution of postoperative medication use by side of extraction (mandibular third molar) in participants (N = 386)

## Discussion

The present study examined the association between postoperative symptom severity and OHRQoL in patients undergoing mandibular third molar extraction. Postoperative symptom severity (PoSSe) and OHIP-14 exhibited a moderate-to-strong positive correlation in our study, with patients with worse OHRQoL reporting more severe postoperative symptoms. These findings align with other studies that have concluded that PoSSe scores significantly predict changes in OHIP-14 scores. However, other factors (impaction position and difficulty index) did not substantially affect postoperative quality of life^12)^.

Our study also showed that gender affects postoperative outcomes. Women reported higher PoSSe and OHIP-14 scores, the latter indicative of poorer OHRQoL. This is in line with the earlier findings that indicate that women were found to complain more of postoperative pain after undergoing mandibular third molar surgery^13)^. However, females had higher OHIP-14 scores than males, indicating poorer OHRQoL. This result contrasts with a study in which males scored higher on OHRQoL than females, suggesting that gender differences in OHRQoL may vary across populations and may also depend on cultural, behavioural, or health-related factors^14)^.

In our research, postoperative symptom severity (PoSSe) scores increased slowly with age, with the highest scores reported by older participants. This is in line with the previous research, which shows that the older the age, the greater the postoperative morbidity in terms of pain, swelling and trismus, despite the mandibular third molar surgery^15)^. We found in our study that OHIP-14 scores increased with age, indicating worse OHRQoL in older subjects. This is consistent with longitudinal findings that older adults, particularly those who lost teeth with age, scored higher on the OHIP-14, an indicator of poor OHRQoL^16)^.

The multiple linear regression analysis revealed that postoperative symptom severity (PoSSe), age, and gender had been highly predictive of OHIP-14 scores. The strongest predictor was PoSSe, indicating that patients with more severe postoperative symptoms reported worse OHRQoL, which aligns with a prospective cohort study^12)^. Older age is associated with slightly higher OHIP-14 scores, consistent with earlier studies reporting higher OHIP-14 scores in older adults who lost teeth^16)^. Conversely, the females in our study scored higher on the OHIP-14. Conversely, prior research reported higher scores in males, indicating that the effects of gender on OHRQoL might not be consistent across populations, possibly due to socio-cultural, behavioural, or population-related differences^14)^. In our research, the side of mandibular third molar extraction was a prominent predictor of OHIP-14 scores, and extractions on a specific side were perceived as having a slightly greater impact on OHRQoL. As far as we know, this aspect has not been considered in earlier research, indicating a new dimension of our findings that warrants further study. The identification of PoSSe as the strongest predictor of OHRQoL has important clinical implications. Early recognition of patients with higher postoperative symptom severity may allow clinicians to implement targeted pain control strategies, enhanced patient counselling, and closer postoperative monitoring, thereby improving recovery outcomes and patient satisfaction

OHIP-14 scores were higher in complex impactions (horizontal/distoangular impactions), suggesting poorer postoperative OHRQoL. This is consistent with past studies that reveal that painful impaction leads to increased postoperative pain, swelling, and mouth opening restrictions, which negatively affect patient morbidity^15)^. On the other hand, the individuals who had undergone a prior extraction experience scored lower in the OHIP-14 total score, possibly because experience with the procedure might reduce anxiety and enhance perceived OHRQoL^17)^.

Lastly, the use of postoperative drugs was associated with a marginally lower OHIP-14 score, suggesting that appropriate management of oral health-related symptoms could positively affect how patients rate their OHRQoL. Other studies have also reported these findings, comparing non- surgical or adjunctive interventions, in which the appropriate use of postoperative care improved immediate OHRQoL^18)^.

In general, these outcomes emphasise the multifactorial predictors of OHRQoL, underscoring the importance of symptom management, patient demographics, and surgical complications in shaping patient-centred outcomes.

### Limitations

Several limitations of this study should be taken into account when interpreting the results. To begin with, the cross-sectional design failed to assess variation in postoperative symptoms and quality of life over time because the data were collected at a single time point. A longitudinal study would have been more informative about the dynamics of the postoperative pain and its elimination. Also, the possibility of selection bias was introduced by convenience sampling, since the sample was collected from selected clinics, both state and private. It was likely not reflective of the broader population of patients receiving mandibular third molar extraction. The study also relied entirely on self-reports, which are subject to recall bias and subjective differences in pain perception, potentially affecting the validity of symptom severity and OHRQoL ratings. The lack of detailed surgical information, such as the amount of bone excised, the form of the flaps, and the surgeon's experience, can affect postoperative symptoms and recovery and is an additional weakness. In addition, the pre-extraction baseline OHIP scores were unavailable, which limits the ability to determine the magnitude of postoperative quality-of-life change. Finally, postoperative medication was self-reported, and compliance or medication accuracy was not objective.

## Conclusion

In conclusion, the study reveals that the severity of postoperative symptoms is associated with OHRQoL among patients who have undergone mandibular third molar extraction. OHRQoL was also worse among patients who experienced higher postoperative pain, highlighting the high functional cost of postoperative symptoms on the quality of everyday life. The factors that were significant predictors of OHRQoL were gender, age, type of impaction, side of extraction, previous extraction experience, and postoperative medication that alleviated burden. The results emphasise the importance of patient-centred care that involves treating the patient holistically during the postoperative phase, a sound approach to pain management, and sufficient surgical planning to enhance recovery and general patient outcomes following the removal of the mandibular third molar.

## Data availability

The datasets generated and analysed during the current study are available from the corresponding author on reasonable request.

## Author contributions

NF and ER conceived and designed the study. KM, AM, and WA contributed to the study design and supervised data collection. SI, FR, and MS were responsible for data acquisition and data entry. ZA and RME conducted statistical analyses and interpreted the data. MS assisted in manuscript drafting and reference organization. MSW served as the corresponding author, coordinated communication, and finalized the manuscript submission. NF and ER critically revised the manuscript for important intellectual content. All authors read and approved the final version of the manuscript.

## Conflicts of interest statement

The authors declare that there are no conflicts of interest.
